# Factors affecting shoulder joint range of motion after breast cancer surgery: a retrospective cohort study in Japan

**DOI:** 10.1007/s00520-025-10260-y

**Published:** 2026-01-15

**Authors:** Soichiro Koyama, Megumi Ozeki, Nariko Nakano, Yuki Okochi, Yuko Kijima, Yohei Otaka

**Affiliations:** 1 Faculty of Rehabilitation, School of Health Sciences, Fujita Health University, Toyoake, Aichi Japan; 2 Department of Rehabilitation Medicine, School of Medicine, Fujita Health University, Toyoake, Aichi Japan; 3 Department of Rehabilitation, Fujita Health University Hospital, Toyoake, Aichi Japan; 4 Department of Breast Surgery, School of Medicine, Fujita Health University, Toyoake, Aichi Japan

**Keywords:** Range of motion, Rehabilitation, Risk factor, Shoulder, Breast cancer

## Abstract

**Purpose:**

To evaluate factors influencing the range of motion (ROM) in shoulder flexion and abduction, which are often compromised by postoperative conditions, including pain, soft tissue damage, and muscle weakness, 1 month after breast cancer surgery in patients undergoing inpatient rehabilitation.

**Methods:**

In this retrospective study, the electronic medical database of a university hospital was searched for patients who underwent inpatient rehabilitation following breast cancer surgery between May 2014 and April 2020. The extracted data included age, sex, affected side, body mass index, number of nodes removed, levels of axillary lymph nodes, type of mastectomy, chemotherapy, radiation therapy, duration of postoperative drain placement, and length of hospital stay after the surgery. Passive shoulder joint ROM was examined before and 1 month after surgery. Multivariable logistic regression was performed to explore the prevalence of and factors associated with the presence of shoulder joint ROM limitations 1 month after surgery.

**Results:**

This study enrolled 258 patients. A total of 210 participants (81.4%) had shoulder ROM limitation after the surgery. Shoulder flexion and abduction ROM decreased after surgery by an average of 31.3° and 35.9°, respectively. Age, number of nodes removed, and total mastectomy were significantly associated with shoulder joint ROM limitation after surgery.

**Conclusion:**

Over 80% of participants had reduced shoulder ROM 1 month after surgery, even after inpatient rehabilitation. We identified age, number of nodes removed, and total mastectomy as risk factors for reduced shoulder ROM, providing useful prognostic information regarding the restriction of passive shoulder ROM after breast cancer surgery.

## Introduction

Breast cancer is the most commonly diagnosed cancer in women worldwide. In 2020, an estimated 2.3 million cases of breast cancer were diagnosed worldwide, and approximately 685,000 women died from the disease. Approximately one-quarter of all cases occurred in East Asia [1]. Treatments for tumor removal and the prevention of recurrence, including surgery, radiotherapy, chemotherapy, and hormonal therapy, have improved patient outcomes; however, these interventions can cause substantial adverse effects. Common adverse effects include upper extremity motion restriction, cardiac complications, bone loss, fatigue, lymphedema, pain, and chemotherapy-induced peripheral neuropathy [2–4].

Breast cancer surgery involving lymph node dissection and excision of the surrounding tissue often impairs shoulder joint range of motion (ROM) [5–10]. In fact, several studies reported that breast cancer treatment is a common cause of prolonged shoulder ROM limitation [7, 9–11]. One study has shown that 60% of women experienced reduced shoulder flexion and abduction after surgery [9]. In the United States of America, factors associated with persistent ROM limitations after surgery included a higher number of positive lymph nodes, older age, and a higher body mass index (BMI) [9]. A study in Israel also reported that decreased shoulder ROM was associated with a higher number of dissected lymph nodes and the size of the dissected tissue [6]. In addition, a study in the Netherlands showed that shoulder external rotation and abduction ROM limitations at 6 weeks after surgery were associated with long-term shoulder ROM limitations [7]. The type of surgery also impacts shoulder ROM recovery after surgery; mastectomy resulted in a significant initial reduction in shoulder abduction ROM compared with breast-conserving surgery [10].

However, there is a paucity of data on the prevalence of shoulder ROM limitation after breast cancer surgery and its related factors in East Asia, including Japan, which has more than 20% of the world's new cases of breast cancer [1]. Differences in the musculoskeletal system around the shoulder joint have been found between racial groups [12–16]. Therefore, factors contributing to changes in shoulder ROM after breast cancer surgery may vary in the East Asian population. This study was aimed at exploring the prevalence of passive ROM limitation in shoulder flexion and abduction and its risk factors in patients 1 month after breast cancer surgery in Japan. A prior study indicated that ROM limitations in shoulder flexion and abduction were frequently observed one month after surgery [9]. Thus, the present study selected this time point for evaluation. From the viewpoint of clinical experience and several studies [9, 17], we hypothesized that the majority of patients would show limitations in passive shoulder flexion and abduction ROM at 1 month after breast cancer surgery. We also hypothesized that these limitations would be influenced by the magnitude of surgical invasiveness and patient characteristics, such as being overweight.

## Methods

### Study design and setting

This retrospective cohort study was conducted at a university hospital in Aichi, Japan. This study was approved by the Medical Research Ethics Review Committee of Fujita Health University (No. HM21-300, approved on 10, 2021) and has been reported in accordance with STROBE guidelines. This study was performed in accordance with the principles of the Declaration of Helsinki. The requirement for informed consent was waived because of the retrospective study design; individuals who did not opt out were included.

### Participants

We enrolled patients who underwent breast cancer surgery between May 2014 and April 2020. The inclusion criteria for the analyses were female patients with breast cancer aged ≥ 18 years, who were treated with a clinical pathway with rehabilitation. The exclusion criteria were a history of upper limb surgery owing to other diseases, occult cancer, and reoperation. In the clinical pathway, patients who underwent breast cancer surgery with axillary lymph node dissection (ALND) were included, and all patients underwent inpatient rehabilitation. Patients who underwent mastectomy with immediate reconstruction were also included. Patients with breast cancer with only sentinel lymph node biopsy (SLNB) were not included in the pathway because this technique is less invasive compared with ALND and is associated with fewer postoperative complications [18, 19]. No radical or prophylactic mastectomies were performed during the data collection period; therefore, these were not included in the analysis data.

### Data collection

Patient records were retrieved from the electronic medical record of a university hospital. The primary outcome was shoulder joint passive ROM, which was examined before and 1 month after breast surgery. Shoulder joint ROM was measured using a standard goniometer according to the guidelines published jointly by the Japan Orthopedic Surgery Society and the Japan Rehabilitation Medical Society in Japan. Measurements were conducted with patients in the supine position. ROM assessments of the shoulder joint were performed by several rehabilitation physicians with clinical experience ranging from 6 to 31 years and several certified occupational therapists with clinical experience ranging from 1 to 14 years. The extracted data regarding the characteristics of patients and surgery-related factors included age (years), affected side (right or left), BMI, number of nodes removed, levels of axillary lymph nodes, type of mastectomy (partial or total), the presence or absence of chemotherapy up until 1 month after breast surgery, the presence or absence of radiation therapy up until 1 month after breast surgery, duration of postoperative drain placement (days), and the length of hospital stay after the surgery (days).

### Rehabilitation

All patients underwent inpatient rehabilitation after breast surgery based on the clinical pathway. In 2014, we designed a clinical path for efficient postoperative inpatient rehabilitation for all patients with axillary lymph nodes who underwent breast cancer surgery at our university hospital in Toyoake, Japan. The rehabilitation program involved a combination of ROM exercises, mild strength training, education on self-care practices, and prescribed home exercises to minimize adverse complications, including the development of lymphedema. The target joints were the shoulder, elbow, wrist, and finger joints, with movement performed in all directions for each joint. Maximum joint angles were generally limited to approximately 90° for shoulder flexion and abduction during the drainage tube placement period. However, full ROM was performed if the patient experienced no pain. The intensity of joint ROM exercises was kept at a level that did not cause pain during the drainage tube placement period. After drainage tube removal, the intensity was adjusted to avoid excessive load on the surgical wound site, aiming for a level where the patient felt a stretch without pain. Strength training primarily used light loads, such as body weight. Education on self-care included proper hygiene and wound care techniques, guidance on performing daily activities to minimize stress on the surgical site, and recommendations to avoid local compression from tight clothing, such as underwear. The detailed rehabilitation program was discussed and determined by the physiatrist and the occupational therapist in charge, according to the patient’s condition. Each rehabilitation session lasted approximately 40 min/day. The frequency of rehabilitation was typically once a day, from the day after surgery to the day before discharge, except on weekends and holidays. The median [interquartile range] sessions during inpatient rehabilitation were 7 (6-10) sessions.

### Statistical analysis

The participants’ characteristics are presented with the usual descriptive statistics. The presence or absence of ROM limitation was determined by subtracting the preoperative measurement from the one-month postoperative measurement of shoulder joint ROM (either flexion or abduction). A decrease in ROM was defined as the presence of ROM limitation, while no change or an increase was defined as the absence of ROM limitation. To assess participant differences based on the presence or absence of passive ROM limitations, we conducted a between-group comparison. Then, we used multivariable logistic regression to determine the factors associated with the presence of ROM limitation. Continuous data are presented as mean (standard deviation [SD]). Categorical variables are expressed as frequencies and proportions. BMI was calculated as weight (kg) divided by height squared (m^2^). BMI was classified into underweight (less than 18.5 kg/m^2^), normal weight (18.5–24.9 kg/m^2^), overweight (25–29.9 kg/m^2^), and obesity (30 kg/m^2^ or more). Comparisons between the participants with the presence or absence of shoulder joint passive ROM limitations, we used the chi-square test to compare categorical variables and Welch’s *t*-test to compare continuous variables. The dependent variable was the presence or absence of shoulder joint ROM limitation (flexion and/or abduction) on the affected side 1 month after breast cancer surgery. The independent variables were age, BMI, number of nodes removed, levels of axillary lymph nodes, type of mastectomy, the presence or absence of chemotherapy up until 1 month after breast surgery, presence or absence of radiation therapy up until 1 month after breast surgery, duration of postoperative drain placement, and length of hospital stay after surgery according to findings from reported studies [5, 7, 9–11, 20, 21]. Odds ratios (OR) and 95% confidence intervals (CIs) for each variable were estimated. Variance inflation factor (VIF) calculations confirmed the absence of concerning multicollinearity (VIF < 10). Statistical analyses were performed using Stata/SE (version 19.0; StataCorp., College Station, TX, USA). Statistical significance was set at *P* values < 0.05.

## Results

**Fig. 1:**
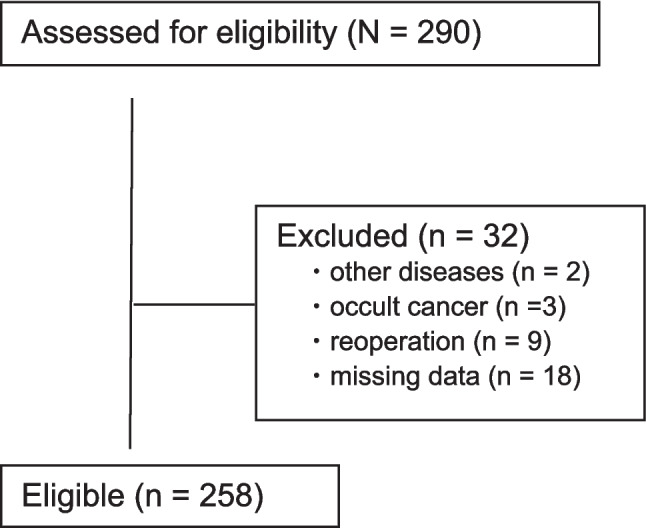
Flow chart of the retrospective cohort study from the database of a clinical pathway at a university hospital from May 2014 to April 2020

**Fig. 2:**
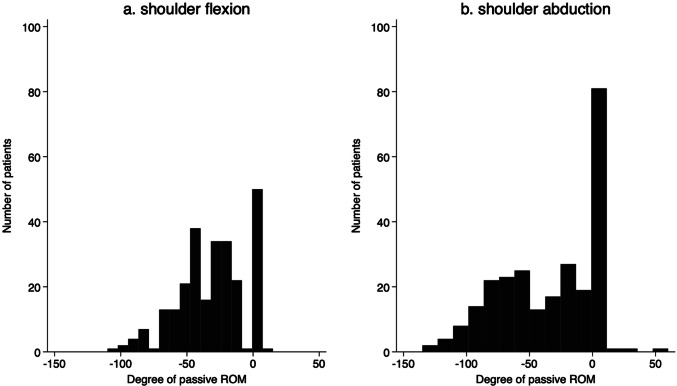
Histogram of shoulder joint range of motion (ROM) limitation. **a** ROM in the shoulder joint at flexion. **b** ROM in the shoulder joint at abduction. In both panels, the horizontal axis shows the change in joint ROM before and 1 month after surgery. The vertical axis is the number of patients. Zero indicates no change in joint ROM from preoperatively to 1 month postoperatively, and negative values indicate joint ROM limitations at 1 month postoperatively

Figure 1 presents a flow chart of the study. A total of 258 patients who underwent breast cancer surgery were enrolled in this study. Table 1 presents the participants’ characteristics. The mean age (SD) at the time of surgery was 56.3 (13.0) years. The mean BMI (SD) at the time of surgery was 23.2 (3.8) kg/m^2^. Histograms of the pre-post shoulder joint ROM difference after 1 month surgery are shown in Fig. 2. A total of 207 participants had reduced passive ROM in shoulder flexion, and 174 participants had reduced passive ROM in shoulder abduction. The shoulder flexion and abduction ROM decreased after surgery by an average of 31.3° (SD 24.6°) and 35.9° (SD 37.0°), respectively.

**Table 1:** Participants’ characteristics (*n* = 258)

Variable	
Age, years, mean (SD)	56.3 (13.0)
Affected side, *n* (%)	
Right	129 (50.0)
Left	129 (50.0)
Body mass index, kg/m^2^, mean (SD)	
< 18.5	29 (11.2)
18.5–24.9	143 (55.4)
25.0–29.9	78 (30.2)
≥ 30.0	8 (3.1)
Nodes removed, *n*, mean (SD)	15.9 (6.4)
Levels of axillary lymph nodes, *n* (%)	
Level I	127 (49.2)
Level II	123 (47.7)
Level III	8 (3.1)
Type of mastectomy, *n* (%)	
Partial	38 (14.7)
Total	220 (85.3)
Chemotherapy, *n* (%)	
No	122 (47.3)
Yes	136 (52.7)
Radiation therapy, *n* (%)	
No	243 (94.2)
Yes	15 (5.8)
Drain placement duration, days, mean (SD)	9.8 (3.9)
Length of hospital stay after surgery, days, mean (SD)	11.6 (5.1)

Categorical variables are presented as numbers and percentages, and continuous variables are presented as means and standard deviations (SD)

Table 2 compares the participants’ characteristics between the participants with and without passive ROM limitations after the surgery in shoulder joint flexion and/or abduction. There were 210 patients with limitations in shoulder flexion and abduction ROM at 1 month after surgery (81.4%). Patients with shoulder ROM limitation were significantly older (*P* = 0.024) than participants without ROM limitation.

**Table 2:** Comparison of characteristics between participants with and without shoulder joint range of motion limitation

Variable	No ROM limitation (*n* = 48)	ROM limitation (*n* = 210)	*P* values
Age, years, mean (SD)	52.4 (12.8)	57.1 (12.9)	0.024
Affected side, *n* (%)			
Right	20 (41.7)	109 (51.9)	0.201
Left	28 (58.3)	101 (48.1)	
Body mass index, kg/m^2^, mean (SD)			
< 18.5	5 (10.4)	24 (11.4)	0.964
18.5–24.9	27 (56.2)	116 (55.2)	
25.0–29.9	14 (29.2)	64 (30.5)	
≥ 30.0	2 (4.2)	6 (2.9)	
Number of nodes removed, *n*, mean (SD)	14.3 (6.2)	16.3 (6.5)	0.050
Levels of axillary lymph nodes, *n* (%)			
Level I	20 (41.7)	107 (51.0)	0.141
Level II	28 (58.3)	95 (45.2)	
Level III	0 (0.0)	8 (3.8)	
Type of mastectomy, *n* (%)			
Partial	11 (22.9)	27 (12.9)	0.076
Total	37 (77.1)	183 (87.1)	
Chemotherapy, n (%)			
No	24 (50.0)	98 (46.7)	0.676
Yes	24 (50.0)	112 (53.3)	
Radiation therapy, *n* (%)			
No	45 (93.8)	198 (94.3)	0.886
Yes	3 (6.2)	12 (5.7)	
Drain placement duration, days, mean (SD)	10.1 (4.3)	9.7 (3.8)	0.581
Length of hospital stay after surgery, days, mean (SD)	11.3 (4.7)	11.6 (5.2)	0.662

*ROM* range of motion, *SD* standard deviation

Table 3 presents the results of the multivariable logistic regression analysis. Level III axillary lymph nodes were excluded as an explanatory factor because all eight participants with level III axillary lymph nodes had limited passive ROM. The final multivariable logistic regression model included age, BMI, number of nodes removed, levels of axillary lymph nodes (levels I and II), type of mastectomy, drain placement duration, and length of hospital stay after surgery. The results showed that older age (OR 1.03; 95% CI 1.01–1.06; *P* = 0.020), number of nodes removed (OR 1.08; 95% CI 1.00–1.15; *P* = 0.020), and total mastectomy (OR 2.62; 95% CI 1.03–6.70; *P* = 0.044) were significantly associated with shoulder joint ROM limitation. There were no factors with a VIF > 10 (1.08 to 3.48).

**Table 3:** Multivariable logistic regression analysis for the presence of shoulder joint range of motion limitation

Variable	Odds ratio	SD	*z*	*P* >|*z*|	95% CI	
Age	1.03	0.01	2.33	0.020	1.01	1.06
Body mass index						
< 18.5	0.91	0.52	−0.16	0.873	0.30	2.82
18.5–24.9	ref					
25.0–29.9	0.95	0.38	−0.13	0.897	0.44	2.06
≥ 30.0	0.41	0.40	−0.92	0.357	0.06	2.76
Number of nodes removed	1.08	0.03	2.33	0.020	1.01	1.15
Levels of axillary lymph nodes						
Level I	ref					
Level II	0.55	0.20	−1.65	0.099	0.27	1.12
Type of mastectomy						
Partial	ref					
Total	2.62	1.25	2.02	0.044	1.03	6.70
Chemotherapy						
No	ref					
Yes	1.29	0.46	0.72	0.468	0.65	2.58
Radiation therapy						
No	ref					
Yes	1.10	0.80	0.13	0.894	0.27	4.56
Drain placement duration	0.77	0.12	−1.65	0.099	0.56	1.05
Length of hospital stay after surgery	1.21	0.19	1.26	0.207	0.90	1.64

*SD* standard deviation, *CI* confidence interval

## Discussion

We explored the prevalence of passive ROM limitation in shoulder flexion and abduction and its associated factors 1 month after breast surgery. Most patients had limited shoulder ROM 1 month after surgery, even when undergoing inpatient rehabilitation. Multivariable logistic regression analysis demonstrated that age, number of nodes removed, and type of mastectomy were significantly associated with shoulder joint ROM limitation. The present results support our hypothesis that the magnitude of surgical invasiveness would be associated with the prevalence of passive ROM limitation in shoulder flexion and abduction. However, the hypothesis that being overweight exacerbates ROM limitations was not supported.

In this study, more than 80% of the participants had reduced shoulder flexion and abduction ROM at 1 month postoperatively, at an average of 31.3° (SD 24.6°) and 35.9° (SD 37.0°), respectively, despite undergoing inpatient rehabilitation. We identified one study from the United States of America that investigated limitations in shoulder flexion and abduction ROM at 1 month postoperatively, reporting a 60% prevalence [9]. Regarding the degree of shoulder ROM limitation at 1 month postoperatively, the following have been reported: approximately 20 to 30° for abduction in Australia [21], 6 to 25° for flexion and 9 to 25° for abduction in Israel [22], and 19 to 52° for flexion and 32 to 66° for abduction in Italy [23]. The results of our study showed the range of shoulder ROM limitations at 1 month postoperatively to be within the typical range. Various studies have revealed racial differences in musculoskeletal characteristics, including joint morphology (e.g., size of humeral head [12], acromion length from the glenoid [13], morphometry of coracoid process [14]), muscle mass [15], and strength [16]). However, despite these racial differences in musculoskeletal characteristics, the degree of ROM limitations was generally consistent with previous studies [21–23]. Restricted shoulder joint ROM following breast cancer surgery has been shown to be influenced by the effects of surgical invasion, including postoperative pain, tightness of the thickened scar, and soft tissue adhesions at the surgical site [24–26]. Regarding the impact of surgical intervention on range of motion limitations, the influence of racial differences in musculoskeletal characteristics is considered to be minimal.

Age, number of nodes removed, and total mastectomy were significantly associated with shoulder joint ROM limitation after surgery. Age was associated with shoulder joint ROM limitation, which is consistent with the result of a prior study [9]. ROM limitations associated with age are affected by changes in the musculoskeletal system, such as reduced ligament elasticity and cartilage resilience, and increased advanced glycation end product [27, 28]. In addition, the presence of postoperative complications (such as wound infection and seroma) also increases with age in patients undergoing breast cancer surgery [29, 30]. These age-related factors may have influenced the present result. However, because the present study did not include data on postoperative complications, it is difficult to conclude why age was associated with reduced joint ROM. The number of removed lymph nodes affected shoulder ROM. This finding is consistent with that of prior studies [6, 9, 11]. The reported average numbers of lymph nodes removed in clinical studies involving patients with breast cancer are 16.4 [31], 15.7 [9], and 12.6 [11], which are comparable to the results of the present study. The number of lymph nodes removed reflects the degree of axillary, which we believe had a greater impact on ROM limitation for total mastectomy than for partial mastectomy.

Contrary to our hypothesis and the findings of previous studies, BMI was not associated with shoulder joint limitations. One study in India used a questionnaire to investigate shoulder stiffness in patients 37 months (median) after various breast cancer surgeries (wide local excision, breast-conserving surgery with axillary nodal dissection, modified radical mastectomy) [17]. They reported that patients with a BMI of 25 or higher experienced shoulder stiffness more often than patients with a BMI of 18.5–24.9. Another study in the United States also reported that a BMI of 25 or greater was significantly associated with decreased shoulder joint ROM at 12 months after various breast cancer surgeries (breast conservation therapy, modified radical mastectomy, simple mastectomy) and with different lymph node dissection ranges (none, ALND, and SLNB) [9]. A study conducted in Canada and the United States showed that, considering the extent of axillary lymph node dissection (SLNB alone, ALND after SLNB, or ALND before), overweight patients were 1.5 times more likely to have decreased ROM at 12 months postoperatively compared with patients with a normal BMI [32]. Although the duration of observation and participant characteristics were slightly different in this study, the effect of these factors is unclear. One possible explanation for the different findings in the present study may be related to the fact that all participants in this study underwent inpatient rehabilitation after breast cancer surgery. BMI is also associated with delayed surgical wound healing after breast cancer surgery [33], which may contribute to the development of ROM limitation. On the other hand, rehabilitation promotes blood circulation, thereby increasing the delivery of oxygen and nutrients to the wound area and promoting wound healing [34]. Therefore, rehabilitation may have promoted healing at the surgical site, thereby mitigating the impact of BMI on shoulder joint ROM limitations. Future studies are required to determine how rehabilitation eliminated differences in ROM limitation according to BMI.

All patients in this study underwent rehabilitation. Prior studies have reported that rehabilitation is effective in improving ROM limitations after breast cancer surgery [21, 31, 35–43]. However, the degree of shoulder joint ROM limitation after breast cancer surgery was comparable to that in previous studies [21–23]. Regarding this point, differences between countries in length of hospital stay may have influenced the results. Hospital stays in Japan tend to be longer compared to those in other countries [44]. Prolonged hospitalization would lead to reduced physical activity, potentially contributing to decreased shoulder joint ROM. Therefore, the present result on the degree of shoulder joint ROM limitation may simultaneously reflect both the beneficial effects of inpatient rehabilitation on shoulder joint ROM and the adverse effects associated with reduced physical activity during hospitalization. Therefore, careful interpretation of our findings regarding the degree of shoulder joint ROM limitation is necessary. Alternatively, the amount of inpatient rehabilitation provided for improving joint ROM may have been insufficient. Future comparative trials are needed to confirm the effect of the amount of inpatient rehabilitation on joint ROM limitations after breast cancer surgery.

This study examined the participants’ characteristics and clinical factors associated with shoulder ROM limitation at 1 month post-surgery in patients who underwent inpatient rehabilitation. Prior studies have reported that rehabilitation (e.g., physiotherapy, yoga, and educational interventions regarding postoperative care) is effective in improving ROM limitations [21, 31, 35–43], particularly in the early postoperative period [45]. However, the period between surgery and rehabilitation, frequency, duration, intensity, and timing of evaluation have not been standardized across studies. Further verification of the effectiveness of rehabilitation is needed. Additionally, data on home exercise instruction and self-care education may not be sufficiently objective because they rely on patients’ self-reported adherence. Therefore, future research should verify the effectiveness of rehabilitation to improve shoulder ROM after breast cancer surgery, establish effective interventions, and determine the appropriate evaluation timing to improve shoulder ROM after breast cancer surgery.

### Limitations

The present retrospective cohort study relied on a clinical pathway for rehabilitation at 1 month postoperatively for breast cancer from a single facility in Japan, which limits the generalizability of the results to populations with a different extent of lymph node dissection and presence of rehabilitation. We excluded patients who did not undergo rehabilitation and those who underwent SLNB, which has relatively few postoperative side effects. Moreover, we also did not evaluate patients beyond one month postoperatively. Therefore, when applying these results to a broader population, the risk of both overestimation and underestimation should be considered. Prospective, multicenter, long-term follow-up studies are needed in the future. Another limitation of the present study is the sole focus on shoulder flexion and abduction ROM. Previous literature has indicated that restricted movement may also occur in other planes of motion, such as extension and external rotation [7, 9]. Future prospective research should incorporate measurements of all shoulder joint movements. This comprehensive approach is essential for identifying potential factors that specifically contribute to movement-direction-specific shoulder joint ROM limitations after breast cancer surgery. In the present study, multiple assessors conducted ROM measurements. Therefore, inter-rater variability may have influenced the results. Future studies using the same assessor throughout the study will improve the measurement reliability.

## Conclusion

Greater than 80% of patients had limited shoulder ROM 1 month after breast surgery, even when undergoing inpatient rehabilitation. We found older age, increased number of nodes removed, and total mastectomy to be risk factors for shoulder joint passive ROM limitation at 1 month after breast cancer surgery. These findings provide useful prognostic information.

## Data Availability

The data that support the findings of this study are not publicly available owing to the sensitive nature of the data; however, they are available from the corresponding author upon reasonable request.
